# Low-Intensity Focused Ultrasound-Responsive Phase-Transitional Liposomes Loaded with STING Agonist Enhances Immune Activation for Breast Cancer Immunotherapy

**DOI:** 10.3390/cancers16213657

**Published:** 2024-10-30

**Authors:** Cong Hu, Yuancheng Jiang, Yixin Chen, Ying Wang, Ziling Wu, Qi Zhang, Meng Wu

**Affiliations:** 1 Department of Radiation Oncology, the First Affiliated Hospital of Guangxi Medical University, Nanning 530021, China; hucong@stu.gxmu.edu.cn; 2 Department of Ultrasound, Zhongnan Hospital of Wuhan University, Wuhan 430071, China; 2022283030121@whu.edu.cn (Y.J.); 2018303060054@whu.edu.cn (Y.C.); 2021283030242@whu.edu.cn (Z.W.); zhangqi1437@whu.edu.cn (Q.Z.); 3 Teaching Office, Zhongnan Hospital of Wuhan University, Wuhan 430071, China; zn003936@whu.edu.cn

**Keywords:** ultrasound, Liposomes, STING agonist, breast cancer, immunotherapy

## Abstract

**Background:** Pharmacologically targeting the STING pathway offers a novel approach to cancer immunotherapy. However, small-molecule STING agonists face challenges such as poor tumor accumulation, rapid clearance, and short-lived effects within the tumor microenvironment, thus limiting their therapeutic potential. To address the challenges of poor specificity and inadequate targeting of STING in breast cancer treatment, herein, we report the design and development of a targeted liposomal delivery system modified with the tumor-targeting peptide iRGD (iRGD-STING-PFP@liposomes). With LIFU irradiation, the liposomal system exploits acoustic cavitation, where gas nuclei form and collapse within the hydrophobic region of the liposome lipid bilayer (transient pore formation), which leads to significantly enhanced drug release. **Methods:** Transmission electron microscopy (TEM) was used to investigate the physicochemical properties of the targeted liposomes. Encapsulation efficiency and in vitro release were assessed using the dialysis bag method, while the effects of iRGD on liposome targeting were evaluated through laser confocal microscopy. The CCK-8 assay was used to investigate the toxicity and cell growth effects of this system on 4T1 breast cancer cells and HUVEC vascular endothelial cells. A subcutaneous breast cancer tumor model was established to evaluate the tumor-killing effects and therapeutic mechanism of the newly developed liposomes. **Results:** The liposome carrier exhibited a regular morphology, with a particle size of 232.16 ± 19.82 nm, as indicated by dynamic light scattering (DLS), and demonstrated low toxicity to both HUVEC and 4T1 cells. With an encapsulation efficiency of 41.82 ± 5.67%, the carrier exhibited a slow release pattern in vitro after STING loading. Targeting results indicated that iRGD modification enhanced the system’s ability to target 4T1 cells. The iRGD-STING-PFP@liposomes group demonstrated significant tumor growth inhibition in the subcutaneous breast cancer mouse model with effective activation of the immune system, resulting in the highest populations of matured dendritic cells (71.2 ± 5.4%), increased presentation of tumor-related antigens, promoted CD8+ T cell infiltration at the tumor site, and enhanced NK cell activity. **Conclusions**: The iRGD-STING-PFP@liposomes targeted drug delivery system effectively targets breast cancer cells, providing a new strategy for breast cancer immunotherapy. These findings indicate that iRGD-STING-PFP@liposomes could successfully deliver STING agonists to tumor tissue, trigger the innate immune response, and may serve as a potential platform for targeted immunotherapy.

## Simple Summary

STING agonists face challenges in breast cancer treatment due to their lower accumulation in tumors and rapid clearance from the body, resulting in a short duration of therapeutic effect. Novel phase-transitional liposomes loaded with STING agonists have been developed to overcome these limitations and are specifically designed for low-intensity focused ultrasound (LIFU)-assisted molecular imaging and precise treatment of breast cancer. With the assistance of LIFU, iRGD-modified liposomes (a targeting peptide) undergo a phase transition into microbubbles, leading to enhanced ultrasound molecular imaging of tumors and facilitating breast cancer immunotherapy by releasing STING agonists from the ultrasound-targeted liposomes.

## 1. Introduction

Breast cancer is a common malignant disease that seriously affects human health [1]. Traditional breast cancer treatments have primarily focused on targeting tumor cells using drugs such as trastuzumab and lapatinib [2,3,4]. However, the limited tumor-targeting efficiency of these drugs results in less therapeutic efficacy and presents a significant risk of toxic side effects on healthy tissues [5,6]. Traditional chemotherapy drugs are particularly ineffective against breast cancer, especially solid connective tissue tumors such as triple-negative breast cancer [5,6,7]. Therefore, identifying a method with the potential to enhance the targeted release of therapeutic drugs directly into tumors is crucial for improving breast cancer treatment.

Immunotherapy offers new opportunities for cancer treatment; however, its low response rate and susceptibility to immune-related adverse events (IrAEs) significantly limit its clinical application [8]. The cGAS–STING signaling pathway serves as an intracellular warning system that detects the presence of viral DNA or chromosomal DNA from cancer cells within the cytoplasm [9]. cGAS (cyclic GMP-AMP synthase) can directly recognize DNA from pathogens and catalyze the synthesis of ATP and GTP into cGAMP. This cGAMP then binds to and activates the STING protein, leading to the activation of downstream immune signaling pathways, mediation of the production of type I interferon, and triggering an immune response [10]. STING agonists represent a promising approach for immuno-oncological treatment [11,12]. They activate the interferon gene and the stimulatory factor signaling pathway [13]. They also play a key role in the immune response to viruses and bacteria. During infections, cyclic dinucleotides (CDNs) are generated and bind to STING proteins, triggering their activation, and signaling to the immune system about the presence of a pathogen, leading to the production of pro-inflammatory cytokines that help to combat the invaders. These cytokines activate immune cells, such as natural killer cells, macrophages, and T cells, which gather at the site of infection to eliminate the pathogen. Activating the STING pathway to induce innate immune responses represents a novel immunotherapeutic strategy that can reverse immune suppression in the tumor microenvironment and enhance immune response rates [10,13,14,15,16]. However, STING agonists face several challenges, including poor drug-like properties, unstable metabolism, and off-target side effects. Further, cancer cells possess the ability to evade recognition by the STING pathway by mimicking normal human cells. Furthermore, the non-differential expression of STING proteins in both normal and tumor cells, along with the persistent pharmacological activity of STING agonists, can result in immune-related adverse effects. These factors significantly limit the immunotherapeutic potentials of STING agonists in clinical settings [17]. Therefore, there is an urgent need to develop immunotherapy methods that can specifically activate the STING pathway at the tumor site. Cyclic dinucleotides are key molecules involved in innate immunity in both microorganisms and animals [18,19,20]. STING agonists like cGAMP possess hydrophilic and negatively charged properties that hinder their entry into the cytosol, which is essential for activating the STING pathway [21]. Upon activation, these agonists stimulate the production of type I interferon, which plays a crucial role in activating CD8+ T cells to present tumor-associated antigens [17,18]. However, STING agonists contain two phosphate groups that hinder their ability to cross the cell membrane, while their target molecules reside in the cytoplasm. Therefore, an effective drug delivery system (DDS) is necessary to facilitate their transport into the cytoplasm across the cell membrane. Thus, an effective transport carrier system for delivering STING agonists to tumor cells and immune cells within the tumor microenvironment is needed to enhance the immune system’s capability to recognize and combat cancer cells.

Liposomes are lipid-based vesicles composed of single or multiple concentric bilayers that enclose a water core [21,22]. Due to their unique structure, they can encapsulate both hydrophilic and lipophilic molecules, making them suitable for a wide range of drug delivery applications [21,23]. Liposomes are considered safe nanocarriers because they are composed of biocompatible and biodegradable lipids, such as phospholipids and cholesterol. Moreover, liposomes can be easily functionalized with various functional groups to achieve targeted specificity, prolong their half-life, enhance cellular uptake, and facilitate stimulus-responsive drug release [21,22,23,24,25,26,27]. These beneficial characteristics of liposomes pave the way for their successful clinical application. Several lipid delivery agents are currently in clinical practice, at various stages of clinical trials, or pending approval [27,28]. Beyond their drug delivery potential, lipid delivery agents can also be utilized for diagnostic purposes. Tumor-targeted delivery systems have been developed to precisely target tumors and enhance the concentration of therapeutic drugs within the tumor region. Active targeted therapy has emerged as a promising approach in cancer nanomedicine, facilitating preferential drug accumulation at the tumor site while sparing healthy tissue, ultimately improving treatment efficacy and reducing harmful side effects [28]. Liposomes are typically functionalized with various tumor-specific ligands or antibodies, such as folate, hyaluronic acid, antibodies, and oligonucleotide aptamers, to achieve active tumor targeting. While angiogenesis is a key characteristic of tumors, often resulting in a dense network of blood vessels, some regions within solid tumors, including breast cancer, may lack microvessels, contributing to significantly reduced accumulation of therapeutic drugs. Tumor-homing penetrating peptides can specifically recognize the endothelium of tumor blood vessels and penetrate extravascular tumor tissue [29]. The iRGD peptide, containing the Arg Gly Asp sequence, is widely present in organisms and serves as a recognition site for the interaction between integrins and their ligand proteins [30]. In cancer therapy, iRGD can enhance the effectiveness of chemotherapy drugs and improve their penetration into tumor tissues. For instance, the combination of iRGD with paclitaxel enhances targeted delivery to tumors and improves anti-tumor effects [31]. iRGD also inhibits tumor metastasis by decreasing the spread of tumors, as it influences the processes of tumor cell invasion and metastasis [32]. In tumor blood vessels, two key molecules, neuropilin-1 (NRP-1) and neuropolin-2 (NRP-2), can regulate the vascular permeability of tumor tissue and enhance permeability through the interaction between the C-end rule (CendR) motif and neuromycin [31,33,34,35,36,37]. Studies have shown that NRP-1 is frequently overexpressed in various human tumor types, including breast cancer, melanoma, glioblastoma, leukemia, and others [31,33]. iRGD peptides are targeting peptides that enhance the penetration of tumor vessels and stroma [29,30]. They effectively bind to the surface of cancer cells exhibiting high levels of αv integrin expression, making them valuable for conjugation with imaging agents or anticancer drug formulations for targeted delivery into tumors.

Ultrasound offers several advantages in treatment and diagnostic applications due to its highly controlled, non-invasive, and, most importantly, deeply penetrating nature to tissues for in situ treatment [38,39,40]. After decades of research by clinical experts and researchers, significant advancements have been made in ultrasound molecular imaging [38,39,40,41,42,43]. Similarly, ultrasound contrast agents can serve dual purposes of diagnostic imaging and delivering drugs or genes for therapeutic applications [38,39,41]. Traditional microbubbles, typically measuring a few micrometers in size, significantly enhance ultrasound echo signals and are primarily used for imaging blood. By contrast, nanoparticles smaller than 700 nanometers can pass through the large pores in the vascular system, enabling various imaging and therapeutic applications. However, the nonlinear backscatter of nanoscale bubbles is significantly reduced, resulting in a suboptimal imaging effect for contrast-enhanced ultrasound. Nevertheless, nanoparticles containing perfluorocarbons can transition into microbubbles through phase-change acoustic droplets, thereby improving their imaging potential [34,38,41].

Recent findings indicate that acoustic droplet vaporization (ADV), which involves the transformation of perfluorocarbon (PFC) droplets induced by ultrasonic waves, enhances blood vessel permeability and can lead to tissue damage [44]. Therefore, the ADV technique holds significant promise for delivering nanocarriers, particularly in targeting cancers located in hard-to-reach areas. Ultrasound-targeted microbubble technology (UTMD) is gaining increasing attention from researchers, especially in the tumor treatment field. This technique enhances cell membrane permeability through acoustic evaporation, facilitating the effective transfer of macromolecules into cells. However, UTMD primarily relies on passive targeting, leading to the destruction of microbubbles without effectively penetrating the tumor vasculature, especially when compared to liposomes. To enhance ultrasound imaging and facilitate precise targeted destruction of microbubbles, low-intensity focused ultrasound (LIFU) is frequently used, allowing for imaging and targeted drug release, combined with tumor diagnosis, treatment, and monitoring. LIFU can accurately target tumor tissue and minimize non-specific phase transitions and damage to normal tissues. In contrast to high-intensity focused ultrasound (HIFU), which delivers significant energy, the sound intensity at the focal point of LIFU ranges from 0.4 to 3.2 W/cm^2^, thereby reducing the risk of potential damage to surrounding healthy tissues [41,42,43]. Cavitation with liposomal constructs is a technique that uses the physical effects of ultrasound-induced cavitation to enhance drug delivery and treatment outcomes with liposomes [44,45]. This phenomenon can be harnessed to modify the behavior of liposomes, including size reduction, enhanced drug delivery, the formation of echogenic liposomes, controlled release, and theranostic applications [46,47]. By utilizing the cavitation effect, researchers have improved the efficacy of tumor treatment by combining ultrasound and liposomal doxorubicin (US-L-DOX) [48].

Previous studies have reported the delivery of STING agonists via liposomes for anti-cancer therapy [17,18]. However, these studies did not compare the anti-tumor activity of liposomal cGAMP formulations with that of free cGAMP, leaving the advantages of STING agonist liposomes unclear. They also assessed the anticancer effect of a liposomal formulation in a genetically modified tumor animal model [17,18], whereas our study employed cGAMP liposomes delivered to an unmodified tumor model, which holds greater clinical translational value. Recent studies have advanced the delivery of STING agonists using nanocarriers for tumors. However, these strategies result in only a small fraction of cancer cells or tumor-infiltrating immune cells (approximately 2–10%) absorbing the agonists [26,49], highlighting their limited ability to diffuse through the dense tumor extracellular matrix (ECM) and their tendency to be cleared by the reticuloendothelial system. In our research, we aimed to employ an iRGD-targeted liposome drug delivery system loaded with the phase change material PFP for molecular imaging and precise tumor treatment through LIFU-assisted cavitation with liposomal constructs. The developed nanoliposome particles comprised four components: (1) a liquid core made of perfluoropentane (PFP), a hydrophobic fluorocarbon compound with a boiling point of 29 °C that is widely used in clinical applications; (2) a lipid shell consisting of biocompatible DSPE-PEG2000-iRGD, DPPG, DPPC, and cholesterol; (3) the STING agonist; and (4) the targeting peptide iRGD. With the assistance of LIFU, iRGD liposomes can undergo a phase transition into microbubbles, enabling ultrasound molecular imaging for tumor diagnosis. Furthermore, increased LIFU irradiation can induce an intracellular ‘explosion effect’ through acoustic droplet vaporization, resulting in the release of STING agonists and enhancing immunotherapy for breast cancer.

## 2. Materials and Animals

Perfluoro-n-pentane (PFP, C5F12) was obtained from J&K Scientific Co., Ltd. (Beijing, China). The lipids 1,2-dipalmitoyl-sn-glycero-3-phosphocholine (DPPC), 1,2-distearoyl-sn-glycero-3-PC (DSPC), and cholesterol were supplied by Avanti Polar Lipids (Alabaster, AL, USA). 1,2-distearoyl-sn-glycero-3-phosphoethanolamine-N-[methoxy-(polyethylene glycol)-2000] (DSPE-PEG2000) and DSPE-PEG2000-iRGD were purchased from Shanghai Advanced Vehicle Technology Co., Ltd. (Shanghai, China). The 4T1 cell line was obtained from ATCC (Manassas, VA, USA). MedChemExpress (Monmouth Junction, NJ, USA) provided the 2′,3′-cGAMP sodium and anti-mouse PD-L1 antibody (clone 10F.9G2). The fluorescent dyes, DiI, Dio, and DiR, were acquired from Sigma-Aldrich Chemical (St. Louis, IL, USA). Lastly, BALB/c mice, with an average weight of 15–20 g, were obtained from the Laboratory Animal Center at Wuhan University, Wuhan, China.

## 3. Preparation and Characterization of Liposomes

Thin film hydration and ultrasonic emulsification methods were used to prepare the liposomal formulations [50]. Briefly, DPPC (4 mg), DSPC (1 mg), DSPE-peg2000-iRGD (2 mg), cholesterol (1 mg), and 2′,3′-cGAMP (1 mg) were added to a round bottom flask and dissolved in 5 mL chloroform. The solvent was then evaporated using a rotary vacuum evaporator (Yarong, Shanghai, China) at 52 °C for 1 h, and the thin lipid films were rehydrated with 4 mL of phosphate-buffered saline (PBS). Afterward, 200 μL of PFP was added to the mixture, which was then emulsified using ultrasound at 100 W with 5 s on–off cycles for 5 min. Finally, the solution was centrifuged at 8000 rpm for 5 min at 4 °C to collect the liposome sediment, which was then resuspended three times in 2 mL of PBS solution. The STING-PFP@liposomes were prepared using the same procedure, with the exception that DSPE-PEG (2000)-iRGD was substituted with DSPE-PEG (2000) in the same molar ratio. The size and surface charge of the liposomes were evaluated using dynamic light scattering (DLS) with a Zetasizer (ZEN 3600, Malvern, UK). The morphology of the liposomes was analyzed using transmission electron microscopy (TEM) (H-7500, Hitachi Ltd., Tokyo, Japan). The stability of the liposomes in 37 °C plasma was evaluated under a microscope (Olympus IMT-2). The encapsulation efficiency (EE) of cGAMP within the liposomal formulations was determined using UV–Vis spectrophotometry. Samples were diluted with 75% ethanol, and the concentration of cGAMP was quantified at 252 nm using a UV/Vis spectrophotometer (UV-2600, Shimadzu, Kyoto, Japan). The EE was calculated using the following equation:

EE (%) = amount of cGAMP in liposomes/total amount of cGAMP added × 100%

## 4. Determination of Target-Binding Ability

To evaluate the target-binding ability of the liposomes, 4T1 cells were cultured in confocal dishes to visualize the interactions with the liposomes. The cells were treated with both iRGD-labeled and non-iRGD-labeled liposomes, each tagged with DiI dye, enabling a direct comparison of their binding efficiencies. After a 4 h incubation, the cells were washed three times with PBS to remove any unbound liposomes and subsequently fixed with 4% paraformaldehyde solution for 10 min. Following fixation, the cells were stained with the lipid dyes DiO and DAPI to highlight the cell membrane and to label the cell nuclei. Finally, the cells were examined using a confocal laser scanning microscope (CLSM, Nikon A1R, Tokyo, Japan).

### 4.1. In Vitro Drug Release

The drug release characteristics of the liposomal formulation were investigated using the dialysis bag method. A 2 mL suspension of liposomes containing the same drug (cGAMP) concentration was placed into a dialysis bag with a molecular weight cut-off (MWCO) of 10,000 Da. The bag was then immersed in 40 mL of PBS at pH 7.4, maintained at 25 °C in a water bath with a shaking speed of 100 rpm. At predetermined time points, aliquots of 4 mL of the medium were extracted and replenished with an equal amount of fresh PBS to maintain the sink condition. In the ultrasound (US)-treated group, the liposomes were irradiated with LIFU at 3 W. An LIFU instrument (LMSC051 ACA; Chongqing Haifu Medical Technology Co., Ltd., Chongqing, China) was set up next to the dialysis sac to deliver pulsed wave (PW) irradiation to the liposome solution. The instrument was operated at a pressure of 1.05 MPa, a frequency of 1.0 MHz, a focal length of 1.5 cm, a focus area of 0.4 cm^2^, and a 50% duty cycle for 5 min at intervals of 0, 1, 2, 4, 8, 12, and 24 h. The drug concentration in the medium was determined using a UV–Vis spectrophotometer (UV-2600, Shimadzu, Japan) at 252 nm. Control samples were processed similarly, excluding LIFU exposure. All tests were performed in triplicate.

### 4.2. In Vitro Ultrasound Imaging

An agarose gel model at 3% (*w*/*v*) was prepared to simulate the phase transition process for in vitro US imaging studies. Both B-mode and contrast-enhanced ultrasonography (CEUS) imaging were conducted using a clinical US scanner (Esaote MyLab90, Florence, Italy). In the ADV experiments, an LIFU system was utilized with a frequency of 1.0 MHz, a pressure of 1.05 MPa, a focal distance of 1.5 cm, and a focal spot size of 0.4 cm^2^, set at 3W power with a 50% duty cycle in pulsed wave (PW) mode. Liposomes (800 µL) were introduced into an agarose gel mold, with a degassed water-filled sac placed adjacent to the gel to ensure that the LIFU focus was accurately centered on the liposome solution.

### 4.3. In Vitro Cellular Toxicity Analysis

The cytotoxicity of the liposomes against 4T1 cells was evaluated using the CCK-8 assay. Briefly, the 4T1 cells were inoculated into 96-well plates at a density of 10,000 cells per well, followed by the addition of 100 µL of DMEM medium and incubation for 24 h. After incubation, the cells were treated with liposomes, with a parallel group of untreated cells serving as the control. To evaluate the effect of US on cell survival, the cells were exposed to US at a power level of 3 W for 5 min, delivered from the base of the culture plates. Following the US treatment, the cells were rinsed and incubated with CCK-8 reagent for 2 h at 24, 48, and 72 h intervals to assess the optical density values.

### 4.4. In Vivo Ultrasound Imaging Investigation

All animal experiments were conducted following the procedures established by Wuhan University’s Institutional Animal Care and Use Committee. A tumor xenograft model was developed in 4–6-week-old BALB/C mice. The mice were inoculated subcutaneously in the right flank with 150 µL of 4T1 cell suspension containing approximately 3 × 10^6^ cells. After 12 days of tumor cell inoculation, the mice received an intravenous injection of 150 µL of either PBS (control group) or a liposome suspension via the tail vein. Subsequently, ultrasound imaging was performed using both B-mode and CEUS-mode to detect the occurrence of ADV following ultrasound exposure.

### 4.5. In Vivo Anti-Tumor Efficacy Assessment

When the tumor reached an average volume of 150 mm^3^ (12 days post-inoculation), the tumor-bearing mice were randomly divided into five groups (*n* = 5): PBS (control), US, STING, liposomes + US, and iRGD-liposomes + US. A volume of 200 µL of either PBS, liposomes, or iRGD-liposomes was administered intravenously via the tail vein every 2 days, with a total of five injections for each treatment group. The body weight of the mice was closely monitored, and the tumor dimensions were measured to calculate the tumor volume using the following formula:

volume = *a* × *b*^2^/2

where ‘*a*’ is the length of the longer diameter and ‘*b*’ is the length of the shorter diameter.

On the 15th day of treatment, the mice were euthanized, and tumor tissues were preserved in 4% formaldehyde. The preserved tissues were then sectioned for immunofluorescence staining, including TUNEL staining to analyze apoptosis. Furthermore, markers such as CD8+, CD49b, and NKG2D were assessed to evaluate the immune response and cell death within the tumor microenvironment. Immunofluorescence analysis was conducted using a confocal laser scanning microscope (CLSM; Nikon A1R, Japan). To calculate the survival curve of the mice (*n* = 10), the PD-L1-treated group received intraperitoneal injections of 100 μg of anti-mouse PD-L1 monoclonal antibody (mAb) every 2 days for a total of five injections. The survival rate for each group was evaluated from the first day of treatment until the day of death.

## 5. Quantifying Dendritic Cell Activation

Mice were administered a dose equivalent to 10 μg of cGAMP via intravenous injection through the tail vein. Eight hours after the injection, the tumor-associated lymph nodes were harvested and processed into a single-cell suspension. To prevent non-specific antibody binding, the suspension was treated with Mouse IgG1κ at a density of 1 × 10^6^ cells/mL. After washing, the cells were incubated with fluorescently labeled antibodies, including anti-mouse CD11b-FITC, anti-mouse CD80-APC, and anti-mouse CD86-PE (BioLegend, San Diego, CA, USA). The fluorescence levels were then measured using a flow cytometer (BECKMAN, San Diego, CA, USA) to assess the activation status of dendritic cells.

## 6. Quantification of Cytokine Concentration in Serum

Blood specimens were obtained 24 h post-treatment. After collection, the samples were allowed to coagulate and were then centrifuged to separate the serum. The serum levels of TNF-α, IFN-β, IL-6, and IFN-γ were quantified using enzyme-linked immunosorbent assay (ELISA) kits (R&D Systems, Basel, Switzerland), following the manufacturer’s instructions to ensure accurate measurements.

## 7. Statistics

All of the experiments were conducted in triplicate and the results are presented as the mean values with their standard deviations (mean ± SD). A *p*-value < 0.05 indicates statistical significance. Statistical analyses were conducted using SPSS 18.0 software.

## 8. Results

### 8.1. Preparation and Characterization of STING Agonist-Loaded Liposomes

**Figure 1:**
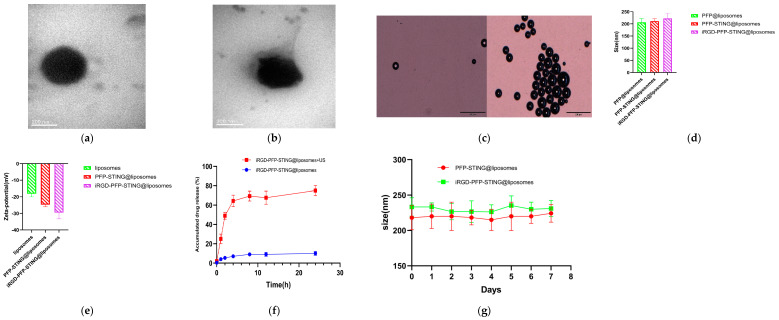
Characteristics of the NPs. Representative TEM images of (**a**) PFP-STING@liposomes and (**b**) iRGD-PFP-STING@liposomes (Scale bar 200 nm). (**c**) Optical microscopy images of LIFU-responsive phase transition of iRGD-PFP-STING@liposomes at 3 min before and after irradiation (Scale bar 20 µm). (**d**) Particle sizes and (**e**) zeta potential of PFP@liposomes, PFP-STING@liposomes, and iRGD-PFP-STING@liposomes. (**f**) cGAMP release curves from iRGD-PFP-STING@liposomes with or without LIFU irradiation. (**g**) The sizes of liposomes and iRGD-liposomes stored in PBS under room temperature conditions for one week.

The transmission electron microscopy (TEM) images confirmed the spherical morphology of the liposomes (Figure 1a,b). The constructed liposomes successfully underwent a phase transition into microbubbles when irradiated with ultrasound, resulting in liposomal membrane rupture and subsequent drug release (Figure 1c,f). The particle size distributions of the control and STING liposomes were 212.25 ± 15.37 nm and 232.16 ± 19.82 nm, respectively, with no significant difference (*p* > 0.05) (Figure 1d). The corresponding zeta potentials for the control and STING liposomes were −21.26 ± 5.64 mV and −27.47 ± 6.25 mV, with no significant difference (*p* > 0.05) (Figure 1e). The liposomes showed good stability after one week of storage in PBS at room temperature in comparison with the freshly prepared samples (Figure 1g). The liposomes were incubated in plasma at 37 °C, and no phase changes were observed under an optical microscope, indicating their stability at this temperature. When stored at 4 °C, the liposomes exhibited minimal spontaneous decomposition, with over 95% remaining intact after one week. The encapsulation efficiency of STING in the liposomal formulation was found to be 42.3%. The measured sizes of the control and STING liposomes were 228.61 nm and 248.92 nm, respectively. The results from the TEM images indicated that the addition of iRGD had minimal impact on the particle size distribution of the liposomes. Furthermore, the size of the liposomes met the criteria for the enhanced permeability and retention (EPR) effect, making them suitable for both passive and active targeting of tumor tissues. Zeta potential is a critical factor in assessing the stability of nanoparticles with a negative value indicating excellent stability, which is advantageous for biopharmaceutical applications.

### 8.2. Target-Binding Ability of STING Agonist-Loaded Liposomes

**Figure 2:**
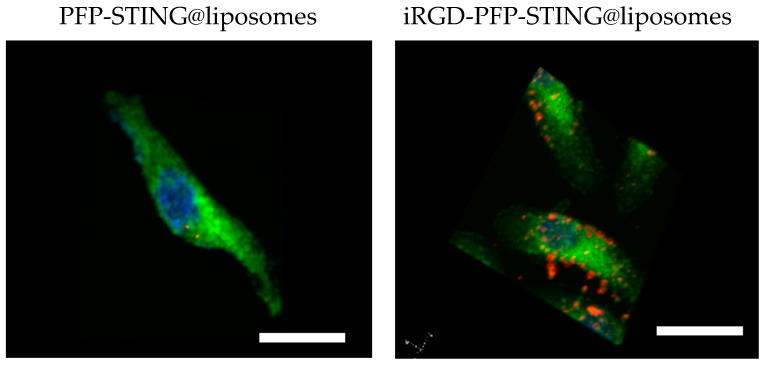
4T1 cells incubated with PFP-STING@liposomes and iRGD-PFP-STING@liposomes. DiI-labeled liposomes (red), DAPI showed blue fluorescence (cell nucleus), DIO-labeled cell membrane (green) Scale bar: 10 µm.

To evaluate the targeting ability of iRGD-labeled liposomes for breast cancer, control liposomes without targeting ligands were also constructed. After 4 h co-incubation with 4T1 breast cancer cells, the cell-targeting efficiency was assessed using a laser confocal microscope. The results demonstrated a significantly increased association of iRGD-targeted liposomes with 4T1 tumor cells. Visualization using CLSM (Figure 2) showed blue fluorescence for the nuclei stained with DAPI and red fluorescence from the labeled nanodroplets in the cytoplasm of the 4T1 cells. The unmodified fluorescent nanodroplets displayed weak intensity, whereas the iRGD-modified liposome group exhibited strong fluorescence within 4T1 cells. This indicated that iRGD modification significantly enhanced liposome internalization into breast cancer cells, thereby potentially improving therapeutic efficacy against breast cancer. In this study, 4T1 cells were co-incubated with DiI-labeled liposomes at 37 °C for 4 h. The results indicated that the targeted liposomes, characterized by a pronounced red fluorescence distribution, exhibited significantly higher uptake compared to the non-targeted group.

#### In Vitro Ultrasound Imaging

**Figure 3:**
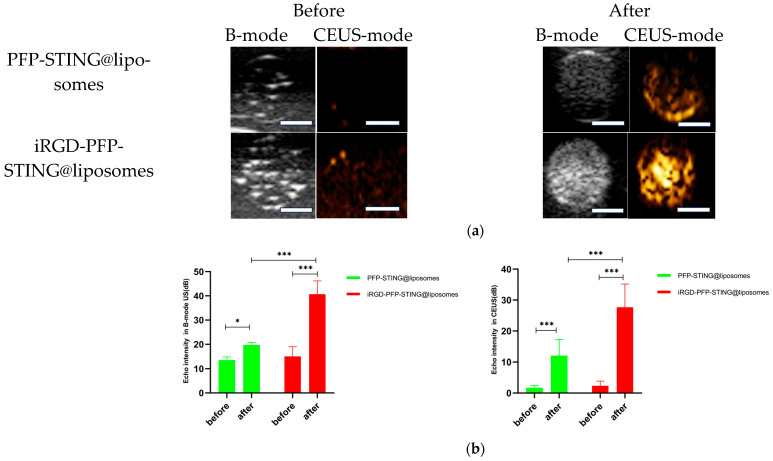
(**a**) B-mode and CEUS-mode ultrasound images of PFP-STING@liposomes and iRGD-PFP-STING@liposomes before and after LIFU irradiation with 3 W for 3 min. Scale bar: 5 mm. (**b**) Echo intensity values of liposomes irradiated using LIFU. Data are represented as the mean ± SEM (n = 3, * *p* < 0.05, *** *p* < 0.001).

The ultrasound imaging of both targeted and non-targeted liposomes pre-ultrasound irradiation revealed no significant difference in US signal intensity between the two groups in both B- and CEUS-modes (*p* > 0.05) as shown in Figure 3. These images were captured using a US system (Esaote MyLab90, Florence, Italy) with the imaging parameters set to MI 0.08, TIB 0.3, and probe type LA523. Two factors were considered to induce the transition of liposomes into bubbles: heating (thermal factor) and ultrasound treatment (mechanical factors). The core of the nanodroplets used in this study was PFP, which has a boiling point of 29 °C at atmospheric pressure. However, according to Laplace pressure principles, droplets encapsulated in lipid shells can stabilize larger droplets and withstand greater pressure, which contributes to an increased evaporation temperature of the droplets [51,52]. This phenomenon suggests that nanodroplets facilitate infiltration into tumor tissues in vivo, enabling effective ultrasound imaging beyond the boundaries of tumor blood vessels. Therefore, they can enhance spatial contrast signals in both B-mode and CEUS-mode. The ADV was fixed at an ultrasound irradiation time of 3 min and an irradiation intensity of 3 W [38]. Following ultrasound irradiation, the liposomes underwent effective vaporization for ultrasound imaging, demonstrating the feasibility of using ADV simultaneously for both imaging and treatment purposes.

### 8.3. Biocompatibility Evaluation of Liposomes In Vitro and In Vivo

**Figure 4:**
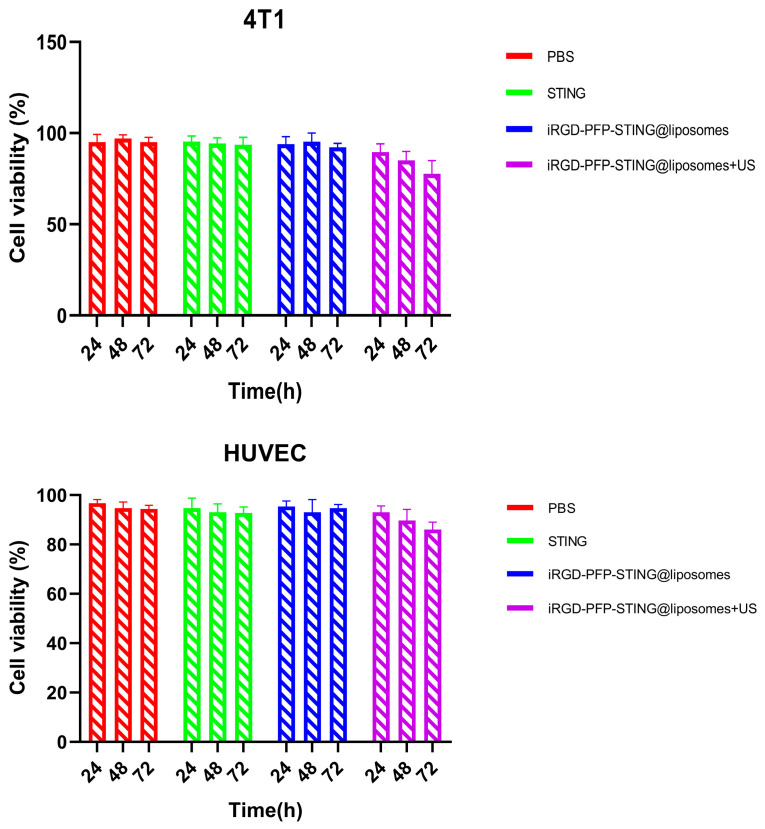
4T1 cell and HUVEC cell viabilities after incubation with PBS, STING, PFP-STING@liposomes, and iRGD-PFP-STING@liposomes+US for 24, 48, and 72 h with or without LIFU irradiation.

The safety and biocompatibility of the liposomes were evaluated in animal models and through cellular assays. Potential cytotoxicity was assessed using the CCK-8 assay in 4T1 and HUVEC cell lines as shown in Figure 4. After 24 h exposure to various concentrations of liposomes, no significant cytotoxic effects were observed. Data analysis across all experimental groups revealed no meaningful differences. Notably, even when treated with a concentrated liposome at a density of 1 × 10^7^/mL, the cell survival rate exceeded 90%, indicating that the liposomes demonstrated non-toxic and biocompatible properties. The in vivo acute toxicity of the liposomes was evaluated in mice followed by histopathological and hematological analyses. Using the PBS treatment group as a blank control, no significant differences were observed in liver function, kidney function, or routine blood test results following liposome injection. The results of in vitro cell testing of targeted and untargeted liposomes showed no significant difference in cell viability compared to the PBS (control) group after ultrasound irradiation, indicating the non-toxic nature of the liposomes. However, when comparing the ultrasound irradiation group to the control group, a decrease in cell viability was observed at the same concentration. This decline was likely due to ADV, which adversely affects cell function. Therefore, the combination of ultrasound treatment and liposomes demonstrated a greater cytotoxic effect.

#### In Vivo Ultrasound Imaging

**Figure 5:**
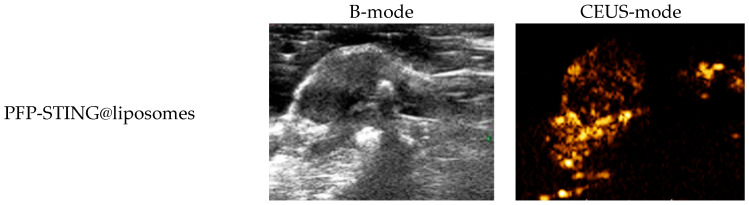

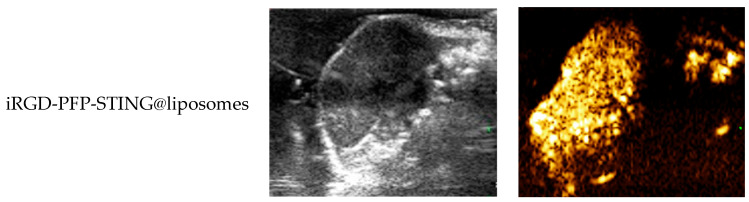
In vivo ultrasound imaging. The US and CEUS imaging pictures of PFP-STING@liposomes and iRGD-PFP-STING@liposomes obtained at 13 min post-injection. LIFU was applied for 3 min in 4T1 breast cancer xenograft model mice.

To evaluate the contrast-enhanced ultrasound effect of the liposomes in vivo, ultrasound imaging experiments were conducted on mice bearing 4T1 breast cancer xenografts. Following the injection of liposomes and subsequent ultrasound irradiation, a significant enhancement of the ultrasound signal was observed in the tumor region in both B-mode and CEUS-mode. Figure 5 illustrates the ultrasound images of the liposomes after destruction. Following 10 min of injection, exposure to LIFU at 3 W/cm^2^ in pulsed wave mode for three minutes resulted in the formation of hyperechoic regions in the tumor, as observed in both B-mode and CEUS-mode images. This finding indicated that phase transition of the nanoliposomes was successfully induced through ADV. In CEUS-mode, the ultrasound signal intensity in tumors treated with targeted iRGD liposomes was higher compared to non-targeted tumors. These results demonstrated that the developed liposomes enabled real-time monitoring and visualization of the tumor treatment area during drug therapy. In addition, the results confirmed that these liposomes exhibited targeted anti-tumor activity and could be effectively employed for ultrasound imaging, representing a promising therapeutic approach for tumor targeting.

### 8.4. Biodistribution of Liposomes

**Figure 6:**
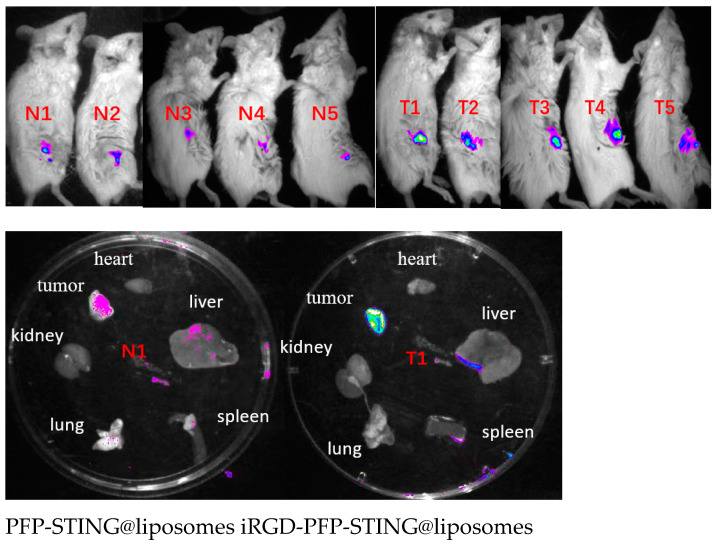
In vivo and different organs (N1 T1) fluorescence imaging of 4T1 tumor-bearing mice.

To evaluate the in vivo targeting ability of the liposomes, DiR-labeled liposomes were administered via tail vein injection, followed by the acquisition of near-infrared fluorescence (NIRF) images in tumor-bearing BALB/c mice. Fluorescence signals were detected shortly after injection, and after 30 min, the tumor signal in the targeted liposome group was significantly higher than that in the non-targeted group (Figure 6).

#### In Vivo Anti-Tumor Efficacy Analysis

**Figure 7:**
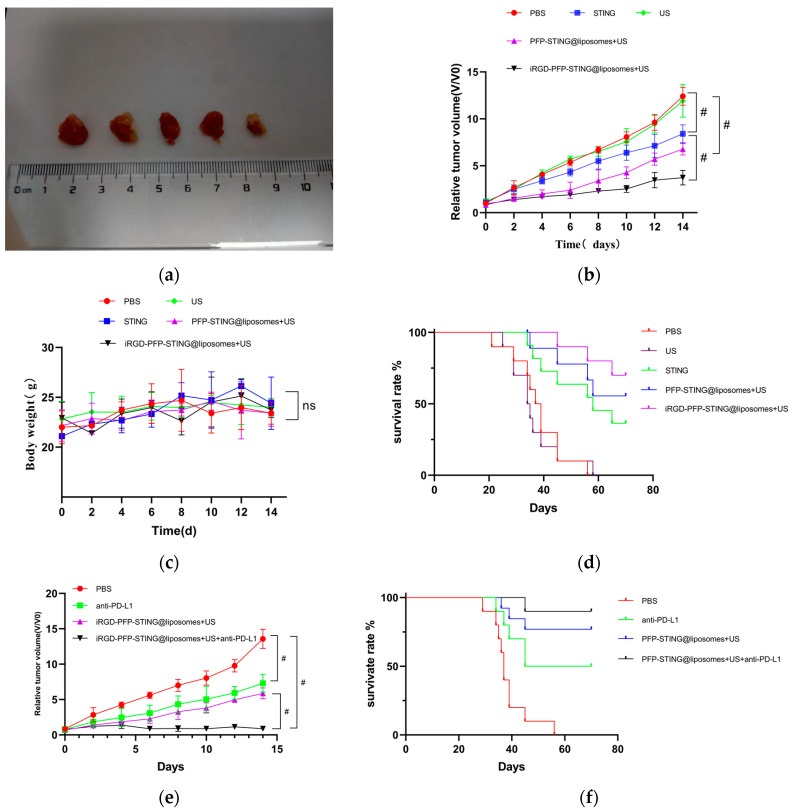
In vivo anti-tumor and therapeutic evaluation. (**a**) Photographs of 4T1 tumor tissues collected at the end of treatment from the PBS, US, STING, PFP-STING @liposomes + US, and iRGD-PFP-STING @liposomes +US groups, respectively. (**b**,**e**) Relative tumor volume changes of the mice after various treatments. (# *p* < 0.001, *n* = 5). (**c**) Body weight changes of the mice after different treatments. (**d**,**f**) Survival curves of mice in different treatment groups over 70 days (*n* = 10).

The anti-tumor effects of targeted and untargeted liposomes and control groups were evaluated in mice implanted with 4T1 cells. The volume of the tumors in PBS-treated mice increased rapidly, whereas the targeted liposome group demonstrated a significant delay in tumor growth throughout the experiment, as illustrated in Figure 7a,b. The growth inhibition rates in the tumor-targeted liposome group and the non-targeted group were 51.26% and 38.74%, respectively. Moreover, the survival rate in the targeted therapy group was significantly higher, with 70% of the mice remaining alive after 70 days, whereas all mice in the PBS control group died (Figure 7d). The targeted in vivo results indicated more effective inhibition of tumor growth in the 4T1 cell-induced mice model. The tumor growth inhibition can be attributed to the passive targeting effect of liposome EPR and the active targeting ability through iRGD. However, the body weights of the mice remained unchanged after the different treatments (Figure 7c). Notably, the combination of iRGD liposomes with anti-PD-L1 therapy (clone 10F.9G2) in PBS (100 μL) stopped tumor growth (Figure 7e) and increased the survival rate of mice from 70% to 90% over 70 days (Figure 7f).

### 8.5. STING Liposomes Change the Immune Status of Breast Cancer

**Figure 8:**
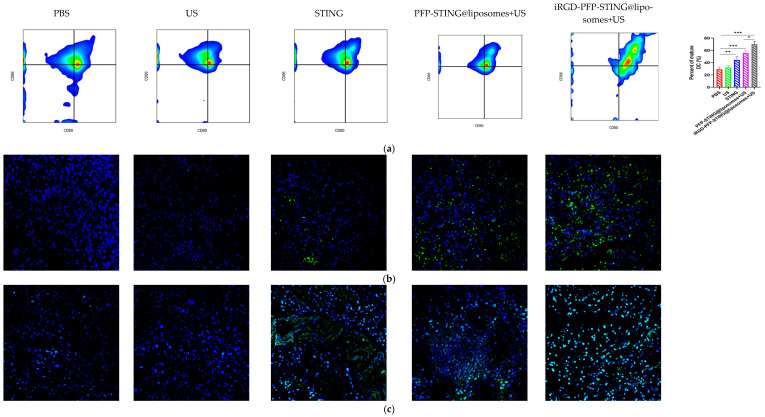

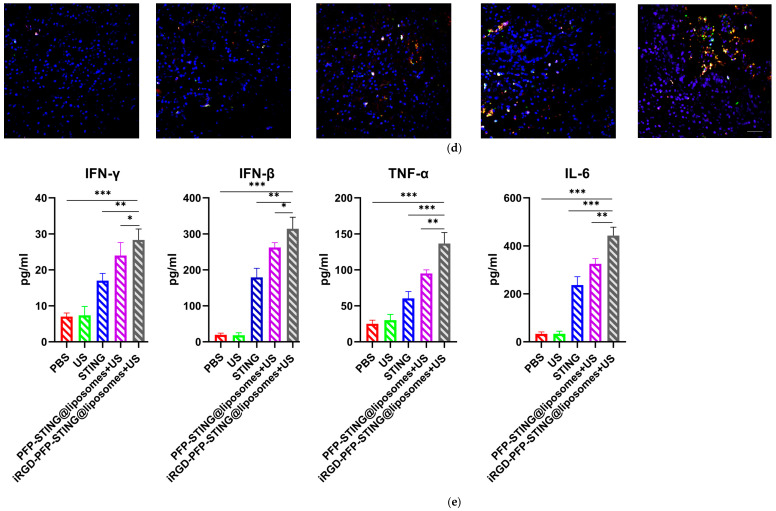
Anti-tumor effects and TME changes in vivo. (**a**) Representative flow cytometry plots of CD80+ or CD86+ among CD11c+ DCs extracted from the tumor sentinel lymph nodes and changes in tumor-infiltrating immune cells in the TME. (**b**) Analysis of CD8+ cells by confocal microscopy. (**c**) TUNEL cells in the TME by confocal microscopy. (**d**) Representative immunofluorescence images showing CD49b (green) and NKG2D (red) expression in tumor tissues harvested from breast cancer tumor-bearing mice after various treatments. Scale bar, 50 μm. (**e**) Cytokine profiles in mice treated with PBS, US, STING, PFP-STING@liposomes +US, and iRGD-PFP-STING@liposomes + US. After 24 h, blood samples were collected. The levels of TNF-α, IL-6, and IFN-γ in serum were measured by ELISA. Data are represented as the mean ± SEM (n = 3, * *p* < 0.05, ** *p* < 0.01, *** *p* < 0.001).

Section staining analysis on the tumors of mice bearing 4T1 breast cancer was performed on the 5th day after five cycles of treatment to study the immune cells. Targeted therapy using ultrasound irradiation led to a significant increase in the infiltration of CD8+ T lymphocytes (CTLs). To further investigate the impact on systemic anti-tumor immunity, flow cytometry analysis was performed on dendritic cells in the tumor-sentinel lymph nodes of mice. Under treatment with ultrasound irradiation, the maturation and activation of DC cells in the lymph nodes of 4T1 tumor-bearing mice in the targeted liposome group increased by 34.2% (Figure 8a). Furthermore, in mice treated with STING agonists combined with liposomes and ultrasound irradiation, the improvements in maturation and activation rates were 12.5% and 24.3%, respectively. These results indicated that ultrasound irradiation of the targeted liposomes enhanced anti-tumor immunity by promoting the infiltration of immune cell populations, particularly CTLs, into tumors (Figure 8b,d). The targeted liposome group showed a significantly enhanced natural immune-killing effect within tumor tissues, as evidenced by the NK cell infiltration analysis in tumor sections. Breast cancer tissues were collected for immunofluorescence analysis, using CD49b and NKG2D as typical markers to assess NK cell activation [53]. The immunofluorescence results showed that the targeted liposome treatment group showed significantly enhanced expression of CD49b (green) and NKG2D (red) in NK cells. The positive proportion of NK cells (CD49b+NKG2D+) was significantly higher than that observed in the other control groups, indicating a considerable expansion and activation of NK cells within the breast cancer tumor microenvironment (Figure 8d). TUNEL staining of tumor slices also indicated the highest proportion of tumor apoptosis in the targeted liposome treatment group, suggesting the excellent tumor cell-killing potential of the targeted liposomes (Figure 8c). The targeted liposomes mice group showed significantly increased serum levels of Th1 cytokines, TNF-α, and IFN-γ, which are typical markers of cellular immunity. Notably, this treatment resulted in marked elevation of IFN-β and IL-6 production, as illustrated in Figure 8e. The stimulation of the STING pathway is known to initiate the synthesis of type I interferons and pro-inflammatory cytokines. Moreover, once activated, NK cells secrete higher levels of TNF-α and IFN-γ. Collectively, these observations suggested that the STING pathway may be a key mediator of NK cell activation induced by liposomal therapy. In conclusion, the evaluation and analysis of immune cells in breast cancer sections indicated that the targeted liposomes developed in this study could transform cold tumors into hot tumors, thereby facilitating effective immunotherapy for breast cancer.

## 9. Discussion

Cold tumors are generally characterized by a lack of sufficient immune cell infiltration within the tumor microenvironment, making them less responsive to immunotherapy due to their limited interaction with the immune system [54,55,56]. By contrast, hot tumors exhibit substantial immune cell infiltration and activity within their microenvironment, making them more susceptible to immunotherapeutic interventions [54]. Their capacity to effectively stimulate the immune system allows for improved targeting and destruction of tumor cells. Converting cold tumors into hot tumors during treatment is a topic of significant interest in cancer research [54,55,56]. Interferon gene stimulating factor (STING) is an immune adaptive protein that can perceive cyclic GMP AMP as a distress signal in response to either self or microbial cytoplasmic DNA [57,58,59,60,61,62]. STING plays a crucial role in regulating the transcription of various defense genes by activating the cGAS–STING signaling pathway [16,58,59,60,61,62]. The cGAS–STING signaling axis is pivotal in cellular recognition of cytoplasmic DNA, playing a key role in shaping immune responses to pathogens, enhancing anti-tumor immunity, and regulating autoimmune reactions. Activation of this pathway triggers the production of type I interferons and various cytokines, facilitating signal transmission to the nucleus. Due to these effects, the cGAS–STING pathway is considered to be a promising therapeutic target for advancing cancer immunotherapy strategies [60,61,62]. STING is widely expressed across various cell types, including cancer cells, and performs multiple functions, such as activating type I interferons, inducing autophagy, and triggering apoptosis [16,58,63]. The increase in IFN-γ levels in STING-LNP leads to an increase in NK expression [59]. Natural killer (NK) cells may serve as vital effector cells for eliminating breast cancer. In this study, we developed a novel targeted liposomal drug delivery system for breast cancer immunotherapy to convert cold breast tumors into hot tumors. The developed targeted cGAMP liposomes effectively activated NK cells, enhanced CD8+ T cell infiltration, and demonstrated a significant anti-tumor effect in a breast cancer mouse model.

Drug delivery systems for the treatment of tumors such as breast cancer still face many challenges [58,62,63]. In the case of solid tumors, the effectiveness of anti-cancer drugs depends on overcoming several challenges in drug transport, including targeting the tumor site while minimizing accumulation in healthy organs and traversing a dense layer of blood vessel walls to reach the cancerous tissue, as some cancer cells are located far from blood vessels [22,23,24]. Similarly, anti-cancer drugs must cross the cell membrane to enter the interior of the cells and exert their anti-cancer effects. Currently, there is no effective method to proactively transport drugs to these areas. The efficient and targeted delivery of anti-tumor drugs into human tumor tissues, which enables controlled drug release and improves drug bioavailability and cellular uptake, is a prominent research topic [35,63]. Ultrasound-mediated liposomal drug delivery systems are widely used currently in both clinical and experimental settings and have demonstrated remarkable results [42,43,44,63]. A novel contrast agent designed with a small volume may facilitate molecular ultrasound imaging and precise tumor treatment by traversing endothelial gaps in tumor tissues [42,43]. Liposomes, recognized for their outstanding biocompatibility and ease of modification, are the preferred choice for membrane materials in this context [17,18,27]. The incorporation of polymers such as polyethylene glycol (PEG) into the lipid membranes of liposomes can significantly enhance their stability and ability to evade detection by the immune system. This modification decreases the susceptibility of liposomes to clearance by the reticuloendothelial system, thereby extending their circulation time within the body. In vivo fluorescence imaging in our study revealed that the liposomes could remain in circulation for over 30 min, indicating their potential for extended systemic circulation—an essential factor for ensuring targeted delivery. Furthermore, the biosafety and biocompatibility of these liposomes were rigorously evaluated through both in vitro cellular assays and in vivo animal studies. After incubation with cells and injection in healthy mice, no significant cytotoxicity or acute toxicity was observed. These findings suggested that the liposomes possessed minimal toxicity and demonstrated excellent biocompatibility, highlighting their potential for clinical application as safe drug delivery carriers. The in vivo experiment involved mice with subcutaneous breast tumors. After the injection of the liposomes into the tail vein and local ultrasound irradiation, rapid enhancement of the ultrasound signal was observed in the tumor area, enabling persistent and clear imaging of the tumor. These findings suggested that the synthesized liposomes possessed strong contrast-enhanced imaging potential. Moreover, current research demonstrates effective performance in both imaging modes.

In this study, iRGD was used to achieve drug enrichment in breast cancer tumors. The peptide target iRGD can effectively perform a three-step process to identify target cells, enabling it to overcome the barriers associated with drug transport in solid tumors [30,31,32,64,65]. The iRGD peptide functions through a sequential targeting mechanism for tumors, first by binding to cells expressing specific proteins, followed by initiating proteolytic cleavage to produce CRGDK fragments. These fragments subsequently interact with αvβ3 and αvβ5 integrins. Subsequently, the fragments bind to neuropilin-1, promoting deeper penetration into the tumor [30,31,32]. The iRGD peptide exhibits a low to medium nanomolar binding affinity for αv integrin, surpassing that of conventional RGD peptides, while the CRGDK fragment shows a strong preference for neuropilin-1 over α integrins [66,67,68]. This preferential interaction and increased affinity facilitate the transition of the CRGDK fragment from integrins to neuropilin-1, enhancing tumor infiltration. Due to its precise targeting and strong binding potential, the iRGD peptide effectively delivers imaging agents and chemotherapeutic drugs deep into the tumor, assisting in tumor detection, inhibiting tumor proliferation, and reducing cancer metastasis [30,31]. RGD is a cyclic peptide with the sequence CRGDKGPDC, which is highly expressed in tumor neovascular endothelial cells as well as in many tumor cells, acting as a ligand for the integrin αvβ3 receptor [34,37,69]. The iRGD peptide serves the function of recognition ligand for the integrin αvβ3 receptor, enabling it to actively target and identify cells with high expression of this receptor. Furthermore, iRGD possesses transmembrane peptide properties, allowing for enhanced drug delivery into human cells via NRP-1 receptors and significantly increasing drug concentrations within those cells [29,30,31,32,66]. Phase-change acoustic nanodroplets operate at the nanoscale and, unlike microbubble contrast agents, utilize the EPR effect of tumors to pass through the gaps between tumor endothelial cells, thereby enhancing ultrasound phase-change imaging in the extravascular space of tumors [38,70,71,72]. In this research, we have successfully developed a novel liposome nanoparticle structure that can more effectively target STING agonists for delivery to tumor cells and activate immune response. This nanoliposome structure specifically directs STING agonists toward tumors and releases them only upon reaching the target tumor cells under ultrasound irradiation. However, the liposomes prepared in this study face several challenges. The preparation process is complex, resulting in high production costs, and limitations in production equipment significantly hinder industrial-scale manufacturing. This study was conducted exclusively in mice, and due to the variability of the EPR effect between mice and humans, as well as the diversity and heterogeneity of human cancer types and the complexity of the tumor microenvironment, the results observed in this experiment require extensive validation before being applied to clinical research.

## 10. Summary

In this study, we developed iRGD-targeted drug-loaded liposome complexes that demonstrated strong ultrasound capabilities, substantial drug-loading efficiency, and precise targeting abilities. The results were confirmed through in vitro targeting experiments, demonstrating that iRGD-targeted drug-loaded liposomes actively adhered to the cell surface. Moreover, live imaging of tumor-bearing mice revealed that the iRGD-modified complex significantly enhanced the tumor-targeting efficacy of the drug delivery system. In summary, this study introduces an innovative drug delivery system utilizing phase change material strategies to enhance the anti-tumor efficacy of STING agonists. The results offer a promising immunotherapy approach using liposomes for breast cancer treatment, emphasizing the potential for targeted and specific therapies in this context.

## Data Availability

The data presented in this study are available upon request from the corresponding author.

## References

[1] Siegel R.L., Miller K.D., Fuchs H.E., Jemal A. (2022). Cancer statistics, 2022. CA Cancer J. Clin..

[2] Swain S.M., Shastry M., Hamilton E. (2023). Targeting HER2-positive breast cancer: Advances and future directions. Nat. Rev. Drug Discov..

[3] Andriani L., Ling Y.X., Yang S.Y., Zhao Q., Ma X.Y., Huang M.Y., Zhang Y.L., Zhang F.L., Li D.Q., Shao Z.M. (2024). Sideroflexin-1 promotes progression and sensitivity to lapatinib in triple-negative breast cancer by inhibiting TOLLIP-mediated autophagic degradation of CIP2A. Cancer Lett..

[4] Karcini A., Mercier N.R., Lazar I.M. (2024). Proteomic assessment of SKBR3/HER2+ breast cancer cellular response to Lapatinib and investigational Ipatasertib kinase inhibitors. Front. Pharmacol..

[5] Di Nardo P., Lisanti C., Garutti M., Buriolla S., Alberti M., Mazzeo R., Puglisi F. (2022). Chemotherapy in patients with early breast cancer: Clinical overview and management of long-term side effects. Expert Opin. Drug Saf..

[6] Diez de Los Rios de la Serna C., Boers-Doets C.B., Wiseman T., Radia B., Hammond R. (2024). Early Recognition and Management of Side Effects Related to Systemic Anticancer Therapy for Advanced Breast Cancer. Semin. Oncol. Nurs..

[7] López-Camacho E., Trilla-Fuertes L., Gámez-Pozo A., Dapía I., López-Vacas R., Zapater-Moros A., Lumbreras-Herrera M.I., Arias P., Zamora P., Vara J.Á.F. (2022). Synergistic effect of antimetabolic and chemotherapy drugs in triple-negative breast cancer. Biomed. Pharmacother..

[8] Mellman G.I., Coukos G. (2011). Dranoff, Cancer immunotherapy comes of age. Nature.

[9] Woo S.R., Fuertes M.B., Corrales L., Spranger S., Furdyna M.J., Leung M.Y., Duggan R., Wang Y., Barber G.N., Fitzgerald K.A. (2014). Sting-dependent cytosolic DNA sensing mediates innate immune recognition of immunogenic tumors. Immunity.

[10] Chen Q., Sun L., Chen Z.J. (2016). Regulation and function of the cGAS-STING pathway of cytosolic DNA sensing. Nat. Immunol..

[11] Luo M., Wang H., Wang Z., Cai H., Lu Z., Li Y., Du M., Huang G., Wang C., Chen X. (2017). A STING-activated nano vaccine for cancer immunotherapy. Nat. Nanotechnol..

[12] Tegtmeyer P.K., Spanier J., Borst K., Becker J., Riedl A., Hirche C., Ghita L., Skerra J., Baumann K., Lienenklaus S. (2019). Sting induces early IFN-β in the liver and constrains myeloid cell-mediated dissemination of murine cytomegalovirus. Nat. Commun..

[13] Marcus A., Mao A.J., Lensink-Vasan M., Wang L., Vance R.E., Raulet D.H. (2018). Tumor-derived cGAMP triggers a STING-mediated interferon response in non-tumor cells to activate the NK cell response. Immunity.

[14] Pan B.S., Perera S.A., Piesvaux J.A., Presland J.P., Schroeder G.K., Cumming J.N., Trotter B.W., Altman M.D., Buevich A.V., Cash B. (2020). An orally available non-nucleotide STING agonist with antitumor activity. Science.

[15] Chin E.N., Yu C., Vartabedian V.F., Jia Y., Kumar M., Gamo A.M., Vernier W., Ali S.H., Kissai M., Lazar D.C. (2020). Antitumor activity of a systemic STING-activating non-nucleotide cGAMP mimetic. Science.

[16] Shae D., Becker K.W., Christov P., Yun D., Lytton-Jean A.K.R., Sevimli S., Ascano M., Kelley M., Johnson D.B., Balko J.M. (2019). Endosomolytic polymersomes increase the activity of cyclic dinucleotide STING agonists to enhance cancer immunotherapy. Nat. Nanotechnol..

[17] Miyabe H., Hyodo M., Nakamura T., Sato Y., Hayakawa Y., Harashima H. (2014). A new adjuvant delivery system ‘cyclic di-GMP/YSK05 liposome’ for cancer immunotherapy. J. Control. Release.

[18] Nakamura T., Miyabe H., Hyodo M., Sato Y., Hayakawa Y., Harashima H. (2015). Liposomes loaded with a sting pathway ligand, cyclic di-GMP, enhance cancer immunotherapy against metastatic melanoma. J. Control. Release.

[19] Hanson M.C., Crespo M.P., Abraham W., Moynihan K.D., Szeto G.L., Chen S.H., Melo M.B., Mueller S., Irvine D.J. (2015). Nanoparticulate sting agonists are potent lymph node-targeted vaccine adjuvants. J. Clin. Investig..

[20] Li S., Luo M., Wang Z., Feng Q., Wilhelm J., Wang X., Li W., Wang J., Cholka A., Fu Y.X. (2021). Prolonged activation of innate immune pathways by a polyvalent STING agonist [published correction appears in Nat Biomed Eng. 2021 May 7]. Nat. Biomed. Eng..

[21] Koshy S.T., Cheung A.S., Gu L., Graveline A.R., Mooney D.J. (2017). Liposomal Delivery Enhances Immune Activation by STING Agonists for Cancer Immunotherapy. Adv. Biosyst..

[22] Torchilin V.P. (2005). Recent advances with liposomes as pharmaceutical carriers. Nature reviews. Drug Discov..

[23] Shi J., Kantoff P.W., Wooster R., Farokhzad O.C. (2017). Cancer nanomedicine: Progress, challenges and opportunities. Nat. Rev. Cancer.

[24] Rosenblum D., Joshi N., Tao W., Karp J.M., Peer D. (2018). Progress and challenges towards targeted delivery of cancer therapeutics. Nat. Commun..

[25] Irvine D.J., Dane E.L. (2020). Enhancing cancer immunotherapy with nanomedicine. Nat. Rev. Immunol..

[26] Kuai R., Ochyl L.J., Bahjat K.S., Schwendeman A., Moon J.J. (2017). Designer vaccine nanodiscs for personalized cancer immunotherapy. Nat. Mater..

[27] Wehbe M., Wang-Bishop L., Becker K.W., Shae D., Baljon J.J., He X., Christov P., Boyd K.L., Balko J.M., Wilson J.T. (2021). Nanoparticle delivery improves the pharmacokinetic properties of cyclic dinucleotide STING agonists to open a therapeutic window for intravenous administration. J. Control. Release.

[28] Large D.E., Abdelmessih R.G., Fink E.A., Auguste D.T. (2021). Liposome composition in drug delivery design, synthesis, characterization, and clinical application. Adv. Drug Deliv. Rev..

[29] Kang S., Lee S., Park S. (2020). iRGD Peptide as a Tumor-Penetrating Enhancer for Tumor-Targeted Drug Delivery. Polymers.

[30] Thirumalai A., Girigoswami K., Pallavi P., Harini K., Gowtham P., Girigoswami A. (2023). Cancer therapy with iRGD as a tumor-penetrating peptide. Bull. Cancer.

[31] Jia G., Han Y., An Y., Ding Y., He C., Wang X., Tang Q. (2018). NRP-1 targeted and cargo-loaded exosomes facilitate simultaneous imaging and therapy of glioma in vitro and in vivo. Biomaterials.

[32] Lo J.H., Hao L., Muzumdar M.D., Raghavan S., Kwon E.J., Pulver E.M., Hsu F., Aguirre A.J., Wolpin B.M., Fuchs C.S. (2018). IRGD-guided tumor-penetrating nanocomplexes for therapeutic siRNA delivery to pancreatic cancer. Mol. Cancer Ther..

[33] Lin J., Zhang Y., Wu J., Li L., Chen N., Ni P., Song L., Liu X. (2018). Neuropilin 1 (NRP1) is a novel tumor marker in hepatocellular carcinoma. Clin. Chim. Acta.

[34] Zhu L., Zhao H., Zhou Z., Xia Y., Wang Z. (2018). Peptide-functionalized phase-transformation nanoparticles for low intensity focused ultrasound-assisted tumor imaging and therapy. Nano Lett..

[35] Ruoslahti E. (2017). Tumor-penetrating peptides for improved drug delivery. Adv. Drug Deliv. Rev..

[36] Ding N., Zou Z., Sha H., Su S., Qian H., Meng F., Chen F., Du S., Zhou S., Chen H. (2019). IRGD synergizes with PD-1 knockout immunotherapy by enhancing lymphocyte infiltration in gastric cancer. Nat. Commun..

[37] Cho H.J., Park S.J., Lee Y.S., Kim S. (2019). Theranostic iRGD peptide containing cisplatin prodrug: Dual-cargo tumor penetration for improved imaging and therapy. J. Control. Release.

[38] Cao Y., Chen Y., Yu T., Guo Y., Liu F., Yao Y., Li P., Wang D., Wang Z., Chen Y. (2018). Drug Release from Phase-Changeable Nanodroplets Triggered by Low-Intensity Focused Ultrasound. Theranostics.

[39] Gong Y., Wang Z., Dong G., Sun Y., Wang X., Rong Y., Li M., Wang D., Ran H. (2016). Low-Intensity Focused Ultrasound Mediated Localized Drug Delivery for Liver Tumors in Rabbits. Drug Deliv..

[40] Chang M., Zhang L., Wang Z., Chen L., Dong Y., Yang J., Chen Y. (2024). Nanomedicine/materdicine-enabled sonocatalytic therapy. Adv Drug Deliv Rev..

[41] Rizzitelli S., Giustetto P., Cutrin J.C., Delli Castelli D., Boffa C., Ruzza M., Menchise V., Molinari F., Aime S., Terreno E. (2015). Sonosensitive theranostic liposomes for preclinical in vivo MRI-guided visualization of doxorubicin release stimulated by pulsed low intensity non-focused ultrasound. J. Control. Release.

[42] Rezayat E., Toostani I.G. (2016). A Review on Brain Stimulation Using Low Intensity Focused Ultrasound. Basic Clin. Neurosci..

[43] Liu J., Shang T., Wang F., Cao Y., Hao L., Ren J., Ran H., Wang Z., Li P., Du Z. (2017). Low-intensity focused ultrasound (LIFU)-induced acoustic droplet vaporization in phase-transition perfluoropentane nanodroplets modified by folate for ultrasound molecular imaging. Int. J. Nanomed..

[44] Schroeder A., Kost J., Barenholz Y. (2009). Ultrasound, liposomes, and drug delivery: Principles for using ultrasound to control the release of drugs from liposomes. Chem. Phys. Lipids.

[45] Zhong Y., Zhang Y., Xu J., Zhou J., Liu J., Ye M., Zhang L., Qiao B., Wang Z.G., Ran H.T. (2019). Low-Intensity Focused Ultrasound-Responsive Phase-Transitional Nanoparticles for Thrombolysis without Vascular Damage: A Synergistic Nonpharmaceutical Strategy. ACS Nano.

[46] Richardson E.S., Pitt W.G., Woodbury D.J. (2007). The role of cavitation in liposome formation. Biophys. J..

[47] Nandkishor R., Mayur A., Srushti M., Indrani M., Ujala G., Rahul N., Priti P., Pankaj K.S. (2024). Unveiling multifaceted avenues of echogenic liposomes: Properties, preparation, and potential applications. J. Drug Deliv. Sci. Technol..

[48] Camus M., Vienne A., Mestas J.L., Pratico C., Nicco C., Chereau C., Marie J.M., Moussatov A., Renault G., Batteux F. (2019). Cavitation-induced release of liposomal chemotherapy in orthotopic murine pancreatic cancer models: A feasibility study. Clin. Res. Hepatol. Gastroenterol..

[49] Cheng N., Watkins-Schulz R., Junkins R.D., David C.N., Johnson B.M., Montgomery S.A., Peine K.J., Darr D.B., Yuan H., McKinnon K.P. (2018). A nanoparticle-incorporated STING activator enhances antitumor immunity in PD-L1-insensitive models of triple-negative breast cancer. JCI Insight.

[50] Yang C., Zhang Y., Luo Y., Qiao B., Wang X., Zhang L., Chen Q., Cao Y., Wang Z., Ran H. (2020). Dual ultrasound-activatable nanodroplets for highly-penetrative and efficient ovarian cancer theranostics. J. Mater. Chem. B.

[51] Sheeran P.S., Dayton P.A. (2012). Phase-change contrast agents for imaging and therapy. Curr. Pharm. Des..

[52] Sainaga Jyothi V.G.S., Bulusu R., Venkata Krishna Rao B., Pranothi M., Banda S., Kumar Bolla P., Kommineni N. (2022). Stability characterization for pharmaceutical liposome product development with focus on regulatory considerations: An update. Int. J. Pharm..

[53] Jiang H., Fu H., Min T., Hu P., Shi J. (2023). Magnetic-Manipulated NK Cell Proliferation and Activation Enhance Immunotherapy of Orthotopic Liver Cancer. J. Am. Chem. Soc..

[54] Galon J., Bruni D. (2019). Approaches to treat immune hot, altered and cold tumours with combination immunotherapies. Nat. Rev. Drug Discov..

[55] Zhang J., Huang D., Saw P.E., Song E. (2022). Turning cold tumors hot: From molecular mechanisms to clinical applications. Trends Immunol..

[56] Liu Y.T., Sun Z.J. (2021). Turning cold tumors into hot tumors by improving T-cell infiltration. Theranostics.

[57] Sun S., Lv W., Li S., Zhang Q., He W., Min Z., Teng C., Chen Y., Liu L., Yin J. (2023). Smart Liposomal Nanocarrier Enhanced the Treatment of Ischemic Stroke through Neutrophil Extracellular Traps and Cyclic Guanosine Monophosphate-Adenosine Monophosphate Synthase-Stimulator of Interferon Genes (cGAS-STING) Pathway Inhibition of Ischemic Penumbra. ACS Nano.

[58] Dosta P., Cryer A.M., Dion M.Z., Shiraishi T., Langston S.P., Lok D., Wang J., Harrison S., Hatten T., Ganno M. (2023). Investigation of the enhanced antitumour potency of STING agonist after conjugation to polymer nanoparticles. Nat. Nanotechnol..

[59] Nakamura T., Sato T., Endo R., Sasaki S., Takahashi N., Sato Y., Hyodo M., Hayakawa Y., Harashima H. (2021). STING agonist-loaded lipid nanoparticles overcome anti-PD-1 resistance in melanoma lung metastasis via NK cell activation. J. Immunother. Cancer.

[60] Corrales L., Glickman L.H., McWhirter S.M., Kanne D.B., Sivick K.E., Katibah G.E., Woo S.R., Lemmens E., Banda T., Leong J.J. (2015). Direct activation of STING in the tumor microenvironment leads to potent and systemic tumor regression and immunity. Cell Rep..

[61] Li S., Mirlekar B., Johnson B.M., Brickey W.J., Wrobel J.A., Yang N., Song D., Entwistle S., Tan X., Deng M. (2022). STING-induced regulatory B cells compromise NK function in cancer immunity. Nature.

[62] Chen X., Meng F., Xu Y., Li T., Chen X., Wang H. (2023). Chemically programmed STING-activating nano-liposomal vesicles improve anticancer immunity. Nat. Commun..

[63] Zylberberg C., Matosevic S. (2016). Pharmaceutical liposomal drug delivery: A review of new delivery systems and a look at the regulatory landscape. Drug Deliv..

[64] Xia T., Liu Z., Du Y., Zhang J., Liu X., Ouyang J., Xu P., Chen B. (2024). Bifunctional iRGD-Exo-DOX crosses the blood-brain barrier to target central nervous system lymphoma. Biochem. Pharmacol..

[65] Singh T., Kim T.W., Murthy A.S.N., Paul M., Sepay N., Jeong Kong H., Sung Ryu J., Rim Koo N., Yoon S., Song K.H. (2024). Tumor-homing peptide iRGD-conjugate enhances tumor accumulation of camptothecin for colon cancer therapy. Eur. J. Med. Chem..

[66] Zuo H. (2019). iRGD: A Promising Peptide for Cancer Imaging and a Potential Therapeutic Agent for Various Cancers. J. Oncol..

[67] Isakova A.A., Artykov A.A., Plotnikova E.A., Trunova G.V., Khokhlova V.A., Pankratov A.A., Shuvalova M.L., Mazur D.V., Antipova N.V., Shakhparonov M.I. (2024). Dual targeting of DR5 and VEGFR2 molecular pathways by multivalent fusion protein significantly suppresses tumor growth and angiogenesis. Int. J. Biol. Macromol..

[68] Reynolds A.R., Hart I.R., Watson A.R., Welti J.C., Silva R.G., Robinson S.D., Da Violante G., Gourlaouen M., Salih M., Jones M.C. (2009). Stimulation of tumor growth and angiogenesis by low concentrations of RGDmimetic integrin inhibitors. Nat. Med..

[69] Zhu Y., Arkin G., Zeng W., Huang Y., Su L., Guo F., Ye J., Wen G., Xu J., Liu Y. (2024). Ultrasound image-guided cancer gene therapy using iRGD dual-targeted magnetic cationic microbubbles. Biomed. Pharmacother..

[70] Luo D., Chen Z., Peng Y., Liu C. (2024). IRGD-modified erythrocyte membrane biomimetic temozolomide nanodots for the treatment of glioblastoma. Nanotechnology.

[71] Zhang P., Cao Y., Chen H., Zhou B., Hu W., Zhang L. (2017). Preparation and evaluation of glycyrrhetinic acid-modified and honokiol-loaded acoustic nanodroplets for targeted tumor imaging and therapy with low-boiling-point phase-change perfluorocarbon. J. Mater. Chem. B.

[72] Kalyane D., Raval N., Maheshwari R., Tambe V., Kalia K., Tekade R.K. (2019). Employment of enhanced permeability and retention effect (EPR): Nanoparticle-based precision tools for targeting of therapeutic and diagnostic agent in cancer. Mater. Sci. Eng. C Mater. Biol. Appl..

